# Evaluation Method of the Vibration Reduction Effect Considering the Real Load- and Frequency-Dependent Stiffness of Slab-Track Mats

**DOI:** 10.3390/ma14020452

**Published:** 2021-01-18

**Authors:** Zeming Zhao, Kai Wei, Wenhao Ding, Fang Cheng, Ping Wang

**Affiliations:** 1 Ministry of Education Key Laboratory of High-Speed Railway Engineering, Southwest Jiaotong University, 4th Floor, Civil Engineering Building, Chengdu 610031, China; zemingZhao@my.swjtu.edu.cn (Z.Z.); whDing@my.swjtu.edu.cn (W.D.); FangCheng@my.swjtu.edu.cn (F.C.); wping@home.swjtu.edu.cn (P.W.); 2 School of Civil Engineering, Southwest Jiaotong University, St No. 111, Beiyi Section, Erhuan Road, Chengdu 610031, China

**Keywords:** slab-track mats, dynamic stiffness, preload dependence, frequency dependence, vibration reduction evaluation

## Abstract

The purpose of this research was to investigate and improve the accuracy of the existing slab-track mat (STM) specifications in the evaluation of the vibration reduction effect. The static nonlinearity and dynamic mechanical characteristics of three types of STMs were tested, and then a modified fractional derivative Poynting–Thomson (FDPT) model was used to characterize the preload and frequency dependence. A modified vehicle–floating slab track (FST) coupled dynamic model was established to analyze the actual insertion loss. The insertion loss error evaluated by the frequency-dependent tangent stiffness increased with the increase in STM nonlinearity, and the error obtained by the third preload tangent stiffness was usually greater than that of the second preload. Compared with the secant stiffness, the second preload frequency-dependent tangent stiffness was more suitable for evaluating STMs with high-static–low-dynamics (HSLD) stiffness. In order to reflect the frequency dependence effect and facilitate engineering applications, it is recommended that second preload tangent stiffness corresponding to the natural frequency of the FST be used for evaluation. Furthermore, the insertion loss of the STMs with monotonically increased stiffness decreased as the axle load increased, and the opposite was true for the STMs with monotonically decreased stiffness. The vibration isolation efficiency of the STMs with HSLD stiffness was both stable and better than that of the STMs with monotonic stiffness.

## 1. Introduction

The convenience and energy-savings of urban rail transit (URT) have driven an increase in urban transportation-oriented development (TOD) construction. For instance, 46 cities in China have constructed 7142 km of URT lines, and the fastest operating speed has reached 160 km/h. However, the high density rail transit network and the fast operating speed inevitably cause more obvious environmental vibrations and noise problems [1]. A common measure to decrease noise emissions and vibrations involves the installation of resilient elements in the railway track [2,3], such as rail pads [4], under-sleeper pads (USM) [5], under-ballast mats (UBM) [6], crumb rubber [7], and slab-track mats (STMs) [8,9,10]. The damping pad floating slab track (DPFST), one of the best measures to reduce vibrations, has been widely used as the main track structure type in the vibration reduction demand area of Guangzhou (160 km/h URT).

Optimizing the stiffness of the damping pad to reduce environmental vibration and structural noise has been extensively studied. Vogiatzis and Kouroussis [11,12] established a finite element (FE) model for the DPFST of the Athens Metro to study and improve the vibration reduction effect (VRE) of the track system. Jin [13] combined the vehicle–floating slab-track (FST)-tunnel–soil coupled model and multi-island genetic algorithm to optimize the stiffness of rubber STMs and improve the VRE. Xin et al. investigated the effect of the stiffness of STMs on the dynamic performance of the transition zone between the fixed slab track and the FST [14]. Liang investigated the influence of the DPFST on structure-borne noise characteristics of a long-span steel truss cable-stayed bridge (LSTCB) and found that the DPFST can reduce the overall sound pressure level by 10–13 dB(A) [15]. However, the linear constant Kelvin (KV) model used in the above-mentioned research may not be accurate in evaluating the actual VRE because it cannot reflect its preload dependence and frequency dependence. The relevant results show that the inclusion of the rail pad nonlinearity into the modelling results in up to 6 dB differences in the peak over a frequency range of 0–200 Hz compared to a linear model [16]. Ma et al. also found that the mitigation effectiveness of the FST is influenced by the preload–track interaction behavior in an experimental test [17].

Some theoretical models with a better performance have been widely used to accurately reflect the impact of the polymer’s mechanical properties on the track’s vibration characteristics. Wei et al. described the dynamic viscoelasticity and plasticity of a Vossloh 300 soft rail pad with a modified fractional derivative Zener (FDZ) model and a traditional Berg friction model, and analyzed the influence on the high-speed vehicle–slab-track coupled vibration [18]. Zhu et al. proposed a stochastic fractional derivative (SFD) model for simulating the dynamic behavior of rubber bearings in the FST [19]. Cezary et al. tested the frequency dependence of UBM and used an FDZ model to evaluate its insertion loss in a two-layer reference system [20]. In addition to the more well-known frequency dependence, temperature dependence, and amplitude dependence of the polymer materials [21,22,23,24,25,26,27], the influence of the preload dependence is now being considered. Koroma et al. successively proposed the modified KV model [28] and the Poynting–Thomson (PT) model [29] considering the preload dependence, which fit better with the measured dynamic stiffness of the fastener pad under different preloads [23]. However, the more accurate the model reflects the true characteristics of the material, the more difficult it is to test and characterize, which means that the application is more inconvenient. Various countries have also issued different evaluation methods for STMs to guide actual projects.

In China, the provisional technical regulation TJ/GW 121-2014 [30] indicates that the train operation safety and VRE are evaluated by the secant stiffness of 3–5 Hz within the range of from the dead load of the FST to the superimposed vehicle load. The original intention of the specification is to facilitate testing and application and to homogenize the differences in different preloads to roughly reflect the overall mechanical characteristics. Under the guidance of this specification, the dynamic behavior design of many STMs in China is undesirably getting increasingly closer to linearization. The international standard DIN 45673-7:2010 [31] pays more attention to the mechanical properties under different preloads and describes three dynamic stiffness test methods of STM in the range of 5–30 Hz to evaluate different indicators. The first preloading stiffness under the dead load of STM is used to verify the tuning frequency of the FST, and the second preloading stiffness under the dead load and half of the live load is used to estimate the dynamic stiffening ratio. The insertion loss can be calculated by the third preloading stiffness under the dead load and full live load or the seconding stiffness [31]. Compared with the domestic standard, the test methods of the DIN standard [31] are more comprehensive, but the standard does not provide a clear method of use in specific applications. For example, the standard does not mention the difference between the second preloading stiffness and the third preloading stiffness in the evaluation of the VRE or which frequency stiffness is used for analysis. In conclusion, the tangent stiffness focuses too much on the dynamic mechanical behavior of single preloading and cannot reflect the actual preloading process from small to large and then to small [32], while the influence of the secant stiffness on the vibration reduction evaluation under different axle loads remains to be studied. Hence, the evaluation differences of different standard methods should be compared and identified with the accurate VRE calculated by the fractional derivative Poynting–Thomson (FDPT) model so as to improve and optimize the specific application method.

To evaluate the VRE of the STMs conveniently and accurately, we improved the existing standard method by comparing the models that can reflect the actual dynamic characteristics. A modified FDPT model was proposed (Section 2), which can reflect the temperature, preload, and frequency dependence. A series of performance tests were carried out on the dynamic properties of the STMs to be used in Guangzhou URT (Section 3.1 and Section 3.2), and the dynamic performance was characterized by the modified FDPT model (Section 3.3). Next, an improved vehicle–FST coupled dynamic model by combining the FDPT model was established to calculate the accurate VRE of STM (Section 4). Finally, the differences between the different evaluation methods (including secant stiffness in the service load range, tangent stiffness under the second and third preload, and actual preload- and frequency-dependent stiffness) were compared and analyzed (Section 5). This research can help optimize the existing evaluation methods and evaluate the VRE simply and accurately.

## 2. Modified FDPT Models

To describe pronounced nonlinear behavior depending on the preload and frequency, the modified KV [28] and PT [29] viscoelastic models are successively used to make empirical approximations for both the static and dynamic stiffnesses of the elastic elements. Fractional calculus can better describe the memory and heredity of various materials and processes, and has been widely used in nonlinear dynamics problems [33,34]. Therefore, we propose a modified FDPT model that can theoretically characterize the dynamic characteristics of STM with the temperature, preload and frequency.

**Figure 1:**
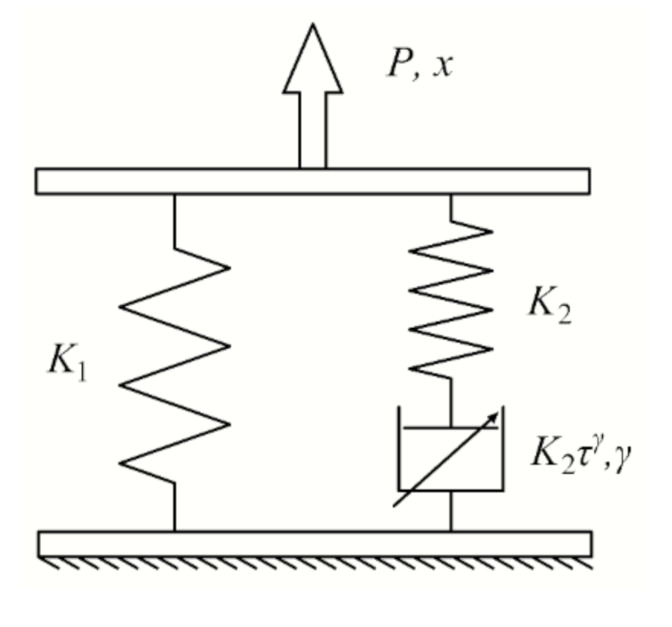
Modified fractional derivative Poynting–Thomson (FDPT) model for slab-track mats (STMs).

The modified FDPT model is shown in Figure 1, where the acting force is *P* and the corresponding displacement is *x*. This model can be divided into two parts: the elastic spring part (*K*_1_) and the fractional derivative Maxwell (FDM) part consisting of the spring (*K*_2_) and Abel dashpot (*K*_2_*τ^γ^*) in series. In this section, the classical FDPT model is first transformed into a temperature-dependent modified model, and then the preload-dependent nonlinear static stiffness and the frequency-dependent dynamic stiffness are respectively derived.

The time-domain constitutive equation of the classic FDPT model and the complex stiffness obtained by Fourier transform (FT) are as follows:

(1) $$P(t)+p_{1}\frac{d^{\gamma }}{dt^{\gamma }}P(t)=q_{0}x(t)+q_{1}\frac{d^{\gamma }}{dt^{\gamma }}x(t)$$

(2) $$K^{*}(i\omega )=\frac{K_{1}+(K_{1}+K_{2}){\left(i\omega \tau \right)}^{\gamma }}{1+{\left(i\omega \tau \right)}^{\gamma }}$$

where *τ* is the relaxation time of the linear viscoelastic material and *γ* is the derivative order. It is worth mentioning that unlike the usual generalized fractional model, the spring stiffness in the FDPT model is considered separately. *K*_1_ and *K*_2_ reflect the static preload-dependent and dynamic frequency-dependent characteristics, respectively.

According to the time-temperature superposition (TTS) [35] of polymer materials and Williams–Landel–Ferry (WLF) equation [36], the temperature-dependent naturalized complex stiffness of K^*^(*iw*,*T*) can be also written as the following equation. The specific calculation procedure can be found in [32].

(3) $$K^{*}(i\omega ,T)=\left(\frac{\rho \cdot T}{\rho_{0}\cdot T_{0}}\right)\times \frac{K_{1}+(K_{1}+K_{2}){\left(i\omega \tau /\alpha (T)\right)}^{\gamma }}{1+{\left(i\omega \tau /\alpha (T)\right)}^{\gamma }}$$

In Equation (3), *T*_0_, *T*, *ρ*_0_, and *ρ*, are the reference temperature, the temperature of interest (in units of K), and the corresponding densities, respectively. *α*(*T*) is the conversion coefficient of the temperatures, in accordance with the following equation:

(4) $$\mathrm{lg}\alpha (T)=\frac{-C_{1}(T-T_{0})}{C_{2}+(T-T_{0})}$$

When the glass transformation temperature (*T*_g_) is regarded as the reference temperature, *C*_1_ and *C*_2_ are 17.44 and 51.6, respectively. If another temperature is selected as the reference temperature, *C*_1_′ and *C*_2_′ at the corresponding reference temperature can be calculated by using Equations (5)–(7).

(5) $${C_{1}}^{\prime }=\frac{C_{1}\times C_{2}}{C_{2}+\Delta }$$

(6) $${C_{2}}^{\prime }=C_{2}+\Delta$$

(7) $$T_{0}=T_{g}+\Delta$$

In the case of static loading, STM is assumed to be purely elastic with a single nonlinear force–displacement curve. The nonlinear static force–displacement relationship can usually be fitted to a polynomial function, and the tangent stiffness *K*_1_ under different preloads can be obtained by derivation in the following form:

(8) $$K_{1}(P)=\frac{dP(x)}{dx}=a_{1}x^{3}+a_{2}x^{2}+a_{3}x^{1}+a_{4}$$

After obtaining the complex stiffness (K^*^) measured by the harmonic excitation and the static nonlinear *K*_1_ obtained by fitting, the stiffness *K*_2_ associated with frequency dependence under different preload conditions can be shown by the following equations:

(9) $$K_{2}(P)={K_{2}}^{\prime }(1+\beta^{\;r})$$

(10) $$\beta =(P-P_{r})/P_{r}$$

where *P_r_* and *K*_2_′ are a reference preload and the corresponding stiffness value, respectively. *β^r^* is an exponential function related to the preload.

The temperature-, frequency-, preload-dependent storage stiffness (*K*′), loss stiffness (*K*″), and loss factor (tan *δ*) can be obtained as follows:

(11) $$K^{\prime }(\omega ,T,P)=\left(\frac{\rho \cdot T}{\rho_{0}\cdot T_{0}}\right)\times \left[K_{1}(P)+\frac{K_{2}(P){\left[\omega \tau /\alpha (T)\right]}^{\gamma }\left[\cos(\gamma \pi /2)+{\left[\omega \tau /\alpha (T)\right]}^{\gamma }\right]}{1+2\cos(\gamma \pi /2){\left[\omega \tau /\alpha (T)\right]}^{\gamma }+{\left[\omega \tau /\alpha (T)\right]}^{2\gamma }}\right]$$

(12) $$K^{\prime \prime }(\omega ,T,P)=\left(\frac{\rho \cdot T}{\rho_{0}\cdot T_{0}}\right)\times \left[\frac{K_{2}(P)\sin(\gamma \pi /2){\left[\omega \tau /\alpha (T)\right]}^{\gamma }}{1+2\cos(\gamma \pi /2){\left[\omega \tau /\alpha (T)\right]}^{\gamma }+{\left[\omega \tau /\alpha (T)\right]}^{2\gamma }}\right]$$

(13) $$\tan\delta (\omega ,T,P)=\frac{K_{2}(P)\sin(\gamma \pi /2){\left[\omega \tau /\alpha (T)\right]}^{\gamma }}{K_{1}(P)+[2K_{1}(P)+K_{2}(P)]\cos(\gamma \pi /2){\left[\omega \tau /\alpha (T)\right]}^{\gamma }+[K_{1}(P)+K_{2}(P)]{\left[\omega \tau /\alpha (T)\right]}^{2\gamma }}$$

## 3. Tests for the Load- and Frequency-Dependent Dynamic Properties of STMs

**Figure 2:**
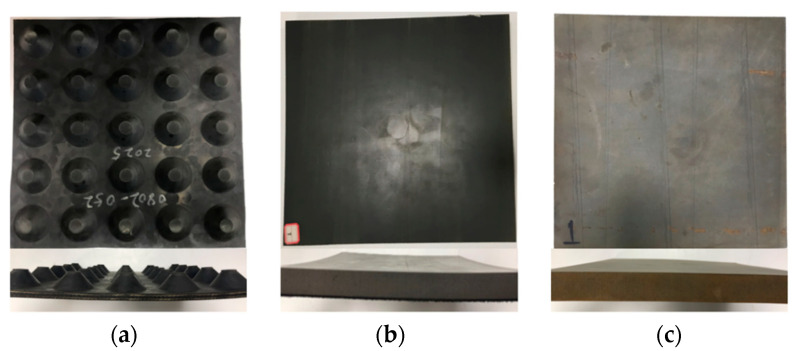
Testing samples: (**a**) STM-I; (**b**) STM-II; (**c**) STM-III.

To compare the sensitivity of different damping pads to the test conditions (e.g., preload and excitation frequency), three kinds of damping pads to be used by Guangzhou URT were adopted as the test objects—see Figure 2. The damping pads are numbered STM-I, STM-II, and STM-III from left to right. The size of all STMs was 300 mm × 300 mm × 25 mm (length × width × installed thickness). STM-I is a rubber-based pad with natural rubber and synthetic rubber, and STM-II and STM-III are both polyurethane-based pads. The tensile strengths of STM-I, STM-II, and STM-III are 15, 0.5, and 0.3 MPa, respectively, and the compression permanent deformations of STM-I, STM-II, and STM-III are 4%, 5%, and 5%, respectively.

**Figure 3:**
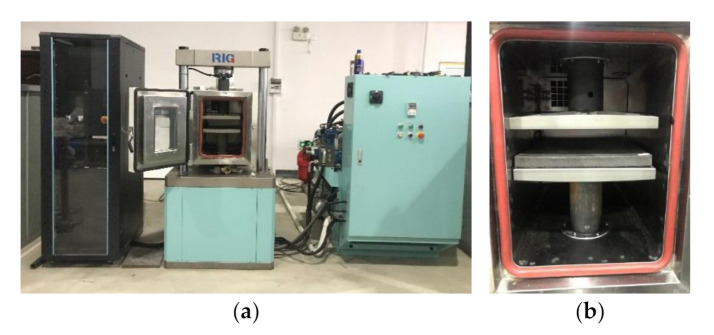
Dynamic test equipment system: (**a**) from left to right are the industrial personal computer, dynamic mechanical tester machine with a temperature control box, and hydraulic power pack; (**b**) from top to bottom are the supporting steel plate, STM, and loading steel plate.

The instrument is shown in Figure 3. The maximum load and maximum frequency of the dynamic mechanical tester are 25 kN and 200 Hz, respectively. The sampling frequencies of the force sensor and displacement sensor are both 5 kHz. The accuracies of the measured force and displacement are 10 N and 2 μm. The harmonic loading mode can be switched between the load control and displacement control. The instrument parameters can meet the harmonic excitation with a particle velocity amplitude of 7 mm/s in the 30 Hz range of the DIN 45673-7:2010 specification [31]. This equipment can not only test the high frequency dynamic mechanical properties of materials but also combine high–low temperature boxes or aging boxes to carry out long-term performance evolution tests.

### 3.1. Test Load Parameter Analysis

**Figure 4:**
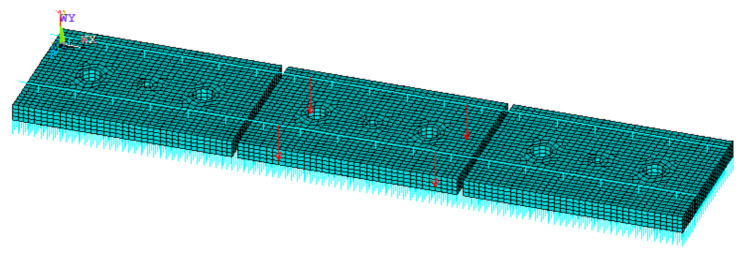
Finite element model of the vehicle–floating slab track (FST).

During the test, the minimum load applied was the FST weight supported within the sample area, and the maximum load was the sum of the minimum load and axle load supported within the sample area when the vehicle ran above the track. It is worth noting that when the speed reaches 160 km/h, the axle load needs to consider a certain dynamic coefficient of 1.3. Three adjacent floating slabs were established by Ansys software (Figure 4), and the load borne by the test sample under the condition of no vehicle load and vehicle load was calculated. The design parameters of the FST and the axle load of the vehicle are shown in Table 1.

The maximum loads obtained by the different STMs’ stiffness were slightly different. The stiffness of the STMs increases from 0.01 to 0.03 N/mm^3^, and the corresponding maximum load increases from 4.0 to 4.2 kN. To facilitate testing, the minimum load and maximum load were determined to be 0.8 and 4.1 kN, respectively.

### 3.2. Test Scheme

A series of experiments were carried out to scientifically test and characterize the dynamic properties of the STMs (see Table 2). Case 1 was conducted to obtain the static nonlinear force–displacement curve of the STMs. Case 2 was based on the standard DIN 45673-7:2010 [31] to test the dynamic stiffness with an interval of 1 kN within the load range of 0–5 kN, and the test frequencies were 5, 10, 20, 40, 80, and 100 Hz. Case 3 was based on the standard TJ/GW 121-2014 to test the dynamic stiffness at different frequencies within the service load range.

### 3.3. Test Results and Model Parameter Fitting

According to the test procedures, the static and dynamic characteristics of the test samples of the STMs were tested.

#### 3.3.1. Static Nonlinearity

**Figure 5:**
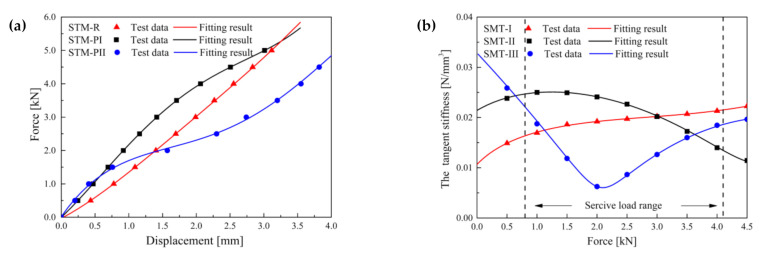
Test and fitting results: (**a**) nonlinear force–displacement curve; (**b**) Nonlinear stiffness–force curve.

Static loading with a nearly zero velocity was carried out on the STMs within the load range of 0 kN to 5 kN at an interval of 0.5 kN. The measured data and fitting results of the nonlinear force–displacement and stiffness–force curve are shown in Figure 5, which confirms the good agreement between Equation (8) and the experimental data.

Although the force–displacement curve of STM-I in Figure 5a is approximately linear, it can be seen from Figure 5b that the stiffness increased slightly. The difference between STM-II and STM-III is that the former decreased gradually within the service load range, while the latter first decreased and then increased. The mechanical properties of STM-III are similar to those of the quasi-zero stiffness isolator with high-static and low-dynamic stiffness. The two ends of the service load range are high in static stiffness to support its own weight and accidental overload, and the main range of the service load is low in dynamic stiffness to achieve vibration isolation. The fitting parameters *a*_1_, *a*_2_, *a*_3_, and *a*_4_ of STM-I, STM-II, and STM-III are shown in Table 3.

#### 3.3.2. Dynamic Stiffness of STMs

**Figure 6:**
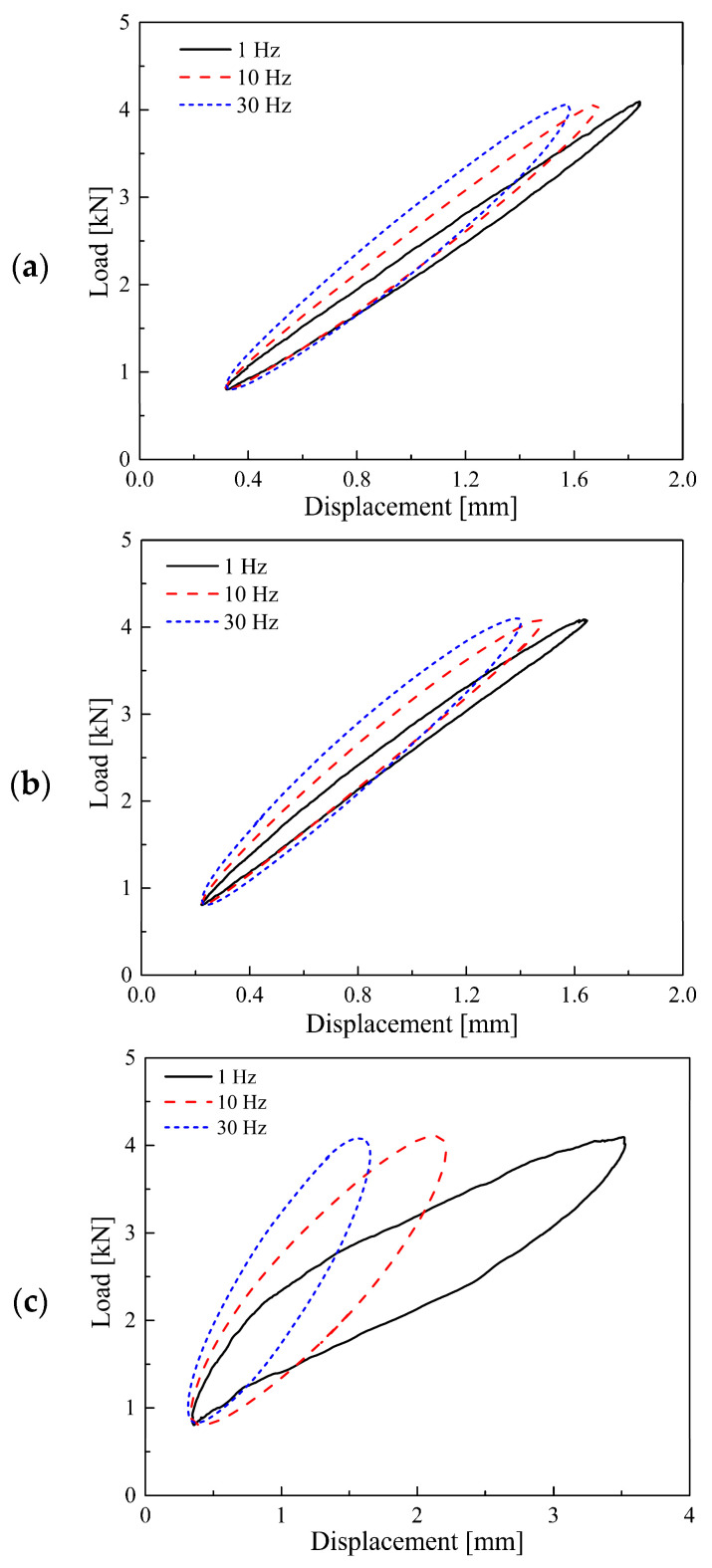
Dynamic hysteresis loop of (**a**) STM-I; (**b**) STM-II; (**c**) STM-III.

In order to avoid repetition, only part of the dynamic hysteresis loops within the service load range are shown, because its load and displacement are larger and the hysteresis loop area is more obvious (see Figure 6).

**Figure 7:**
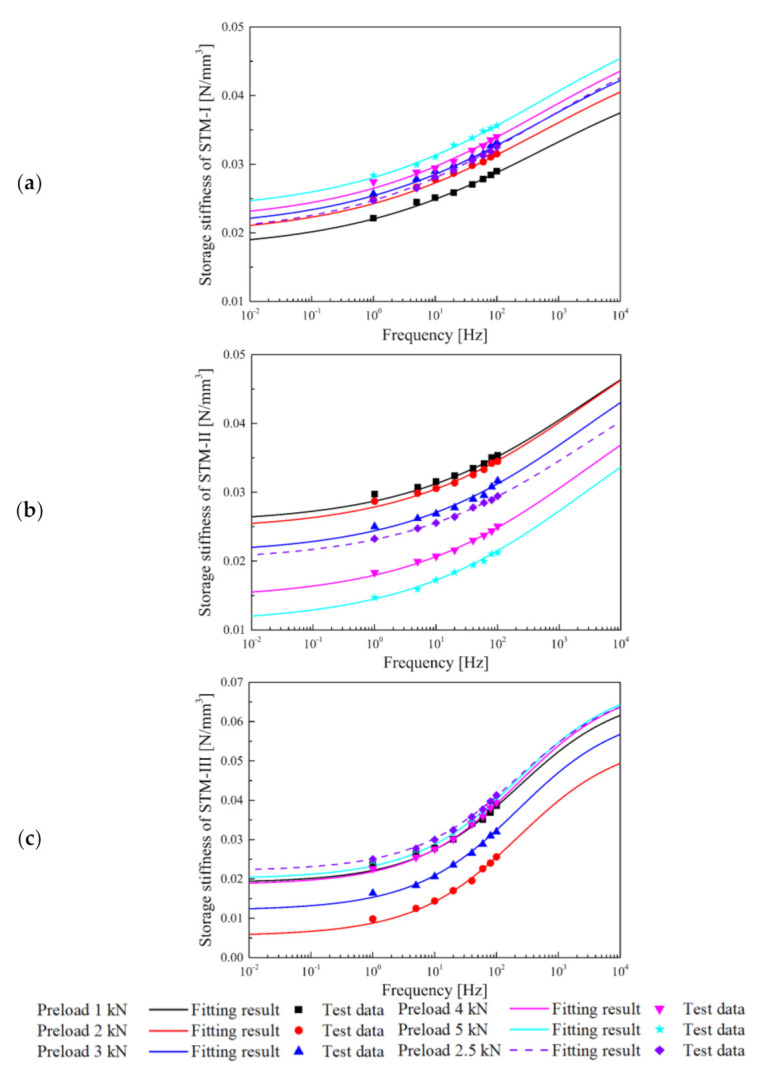
Test data and fitting result: dynamic storage stiffness of (**a**) STM-I; (**b**) STM-II; (**c**) STM-III.

It can be observed that the frequency-dependent stiffness and loss factor of STM-I and STM-II are basically the same, while STM-III not only has obvious frequency-dependent stiffness but also a higher loss factor (that is, a larger dynamic hysteresis loop area). According to these measured hysteresis loops, the stiffness at different preloads and frequencies can be obtained. The test data and fitting results of secant stiffness and tangent stiffness of the STMs are shown in Figure 7.

Under different preloads, the stiffness of the STMs at a frequency approaching 0 is basically consistent with the static stiffness in Figure 5b. Furthermore, the frequency-dependent effect of the same STM under different preload conditions is basically the same. In the range of 1–200 Hz, the stiffness increases in the slopes of STM-I and STM-II (6.81 × 10^−5^ and 6.83 × 10^−5^, respectively) are relatively small. The frequency-dependent effect of STM-III with strong nonlinearity in the service load range is obvious, and the stiffness growth rate is 1.25 × 10^−4^. For STM-III, which has a relatively small static stiffness in the load range of 2–3 kN, a moderately large frequency change effect can also effectively suppress the dynamic displacement of the floating slab. It is worth noting that the secant stiffness of STM-I and STM-II is between the preloaded tangent stiffness of 2 and 4 kN, while the secant stiffness of STM-III is greater than the preloaded tangent stiffness of 1 and 5 kN.

The fitting process under a single preload can be directly calculated using Equation (2), and the stiffness and preload force values with a reference preload of 0.5 kN are selected as *K*_2_′ and *P_r_* in the fitting. The fitting parameters *K*_2_′, *r*, *τ*, and *γ* are shown in Table 4.

**Figure 8:**
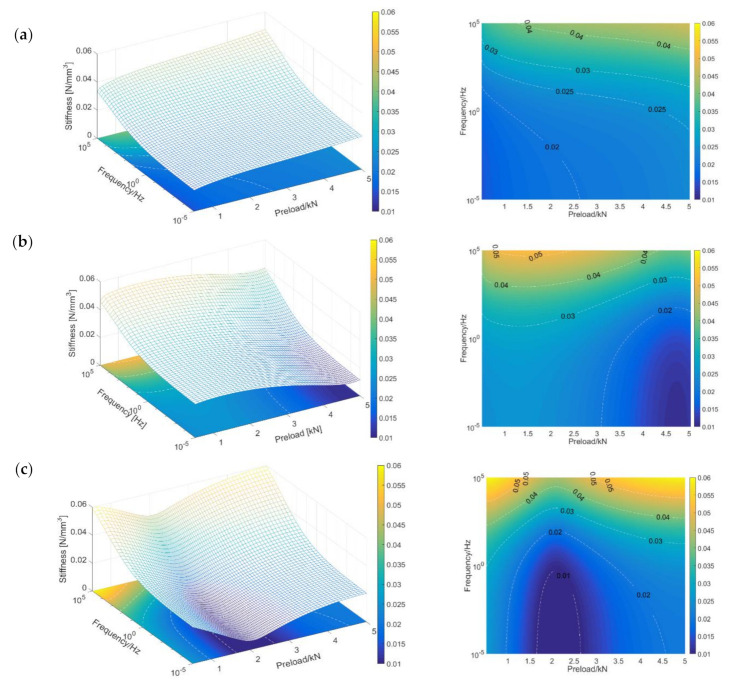
Predicted storage stiffness of STMs under different frequencies and various preloads at 20 °C: (**a**) STM-I; (**b**) STM-II; (**c**) STM-III.

Using the modified FDPT model in Table 4, the preload- and frequency-dependent dynamic performance of the STMs can be easily predicted, as shown in Figure 8. The three-dimensional view and the contour map clearly reflect the difference in nonlinearity and frequency dependence of the different STMs in the service load range.

## 4. Modified Vehicle–DPFST Coupled Dynamic Model Combined with the FDPT Models

**Figure 9:**
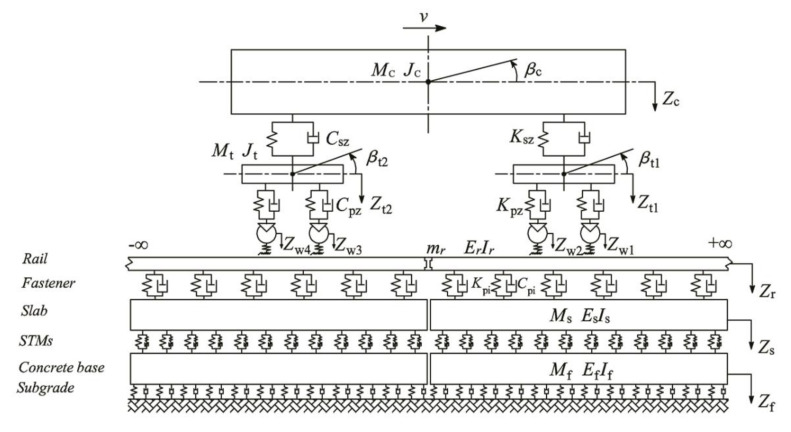
Vertical vehicle–damping pad floating slab track (DPFST) coupled dynamic model.

In order to accurately evaluate the influence of the preload- and frequency-dependent characteristics of the STMs on the VRE of the rail transit system, a vertical vehicle–DPFST coupled dynamic model combined with the FDPT model for the STMs was used to investigate a URT under construction in Guangzhou (see Figure 9). In the dynamics simulation, Chinese CRH6 vehicles, CN60 rail, and prefabricated short floating slabs were used as the research objects.

### 4.1. Vehicle Dynamic Model

The railway vehicle was generally simulated as a multi-rigid-body model with two suspensions. The degrees of freedom mainly included the up and down and nodding movement of a vehicle body, two bogies, and four wheel sets. The differential equations of the vehicle system can be found in [37]. The parameters of the CRH6 vehicle are shown in Table 5. In order to investigate the impact of different axle loads on the VRE in the subsequent calculations, the vehicle body mass was considered to be 25.7 t at no imposed load and 57.2 t at full load.

### 4.2. DPFST Dynamic Model

The DPFST dynamic model consisted of a rail, fasteners, slabs, STMs, concrete bases, and subgrade. The floating slab was simulated as a free beam with discrete supports according to the sample sizes of the STMs. The concrete base was laid on the subgrade, which was simulated as a continuously supported free beam. The fasteners were simply modeled with the Kelvin model, and the STMs were modeled by the FDPT model proposed in this paper.

The vertical vibration equation for the rail can be written as

(14) $$E_{r}I_{r}\frac{\partial^{4}Z_{r}}{\partial x^{4}}+m_{r}\frac{\partial^{2}Z_{r}}{\partial t^{2}}=-\sum_{i=1}^{N_{f}}F_{\mathrm{rs}i}(t)\delta (x-x_{i})+\sum_{j=1}^{4}p_{j}\delta (x-x_{wj})$$

where *m*_r_, *E*_r_ and *I*_r_ are the mass, modulus, and rotational inertia of the rail, respectively; *Z*_r_ is the vertical displacement of the rail; *x*_i_ and *x*_wj_ are the positions of the *i*-th fastener and the *j*-th wheelset, respectively; *N*_f_ is the total number of fastenings; *δ*(*x*) is the Dirac delta function; *F*_rsi_ is the vertical force applied to the rail by the *i*-th fastener, as shown in Equation (14); and *p*_j_ is the vertical force applied to the rail by the *j*-th wheel.

By applying Ritz’s method and normalized shape functions of Euler beams, the partial derivative equation (Equation (14)) can be transformed into the time-dependent differential equations, as follows:

(15) $${\ddot{q}}_{k}(t)+\frac{E_{r}I_{r}}{m_{r}}{\left(\frac{k\pi }{l_{r}}\right)}^{4}q_{k}(t)=-\sum_{i=1}^{N_{f}}F_{fi}(t)Y_{k}(x_{i})+\sum_{j=1}^{4}p_{j}(t)Y_{k}(x_{wj})$$

where *q_k_* (*t*) is the kth generalized coordinate of the rail vertical deflection, *l*_r_ is the simulation length of the rail, and *Y_k_*(x) is the kth orthogonal function, defined as follows

(16) $$Y_{k}(x)=\sqrt{\frac{2}{m_{r}l_{r}}}\sin\frac{k\pi x}{l_{r}}$$

The differential equations of the floating slab supported by the STMs can be written as follows:

(17) $$E_{s}I_{s}\frac{\partial^{4}Z_{s}}{\partial x^{4}}+\frac{M_{s}}{L_{s}}\frac{\partial^{2}Z_{s}}{\partial t^{2}}=\sum_{i=1}^{N_{p}}F_{fi}\left(t\right)\delta \left(x-x_{i}\right)-\sum_{g=1}^{N_{s}}F_{sg}\left(t\right)\delta \left(x-x_{g}\right)$$

where *M*_s_, *E*_s_, *L*_s_ and *I*_s_ are the mass, modulus, length, and rotational inertia of the floating slab, respectively; *N*_p_ and *N*_s_ are the total number of fasteners and STMs on each slab, respectively; and *F*_sg_ is the vertical supporting force applied to the floating slab by the *g*th STM, as shown in Equation (18). The detailed derivation process of the supporting force of the fractional-order model is detailed in Reference [24].

(18) $$F_{sg}(t)=G_{1}Z_{s}(x_{i},t)+G_{2}\sum_{m=1}^{N_{sg}-1}A_{m+1}Z_{s}(x_{i},t-m\Delta t)-G_{3}\sum_{m=1}^{N_{sg}-1}A_{m+1}F_{sg}(t-m\Delta t)$$

in which

(19) $$\left\{\begin{array}{l} G_{1}=\frac{K_{1}(P)+\left[K_{1}(P)+K_{2}(P)\right]\tau^{\gamma }{\left(\Delta t\right)}^{-\gamma }}{1+\tau^{\gamma }{\left(\Delta t\right)}^{-\gamma }}\\ G_{2}=\frac{\left[K_{1}(P)+K_{2}(P)\right]\tau^{\gamma }{\left(\Delta t\right)}^{-\gamma }}{1+\tau^{\gamma }{\left(\Delta t\right)}^{-\gamma }}\\ G_{3}=\frac{\tau^{\gamma }{\left(\Delta t\right)}^{-\gamma }}{1+\tau^{\gamma }{\left(\Delta t\right)}^{-\gamma }} \end{array}\right.$$

where *N_sg_* is the total number of the past integral steps (generally, 160), which makes the calculated support force take into account the loading history process. The fractional parameters *G*_1_–*G*_3_ are functions of *K*_1_(*P*) and *K*_2_(*P*), which makes the FDPT model not only reflect the frequency dependence of the STMs but also consider the influence of the imposed preload. Δ*t* is the integral step and *A_m_* is the *m*th Grünwald–Letnikov coefficient, defined as follows:

(20) $$\begin{array}{c} A_{m+1}=\frac{\Gamma (m-\gamma )}{\Gamma (-\gamma )\Gamma (m+1)} \end{array}$$

Similarly, the dynamic equation of the floating slab can be transformed into a second-order differential equation

(21) $${\ddot{T}}_{n}(t)+\frac{E_{s}I_{s}\beta_{n}^{4}l_{s}}{M_{s}}T_{n}(t)=\sum_{i=1}^{N_{p}}\frac{F_{fi}(t)}{M_{s}}X_{n}(x_{i})-\sum_{g=1}^{N_{s}}\frac{F_{sg}(t)}{M_{s}}X_{n}(x_{g})$$

where *T_n_*(*t*) is the *n*th generalized mode coordinate of the floating slab, and *X_n_* is the nth modal function, defined as follows:

(22) $$\left\{\begin{array}{l} X_{1}=1\\ X_{2}=\sqrt{3}\left(1-\frac{2x}{l_{s}}\right),\\ X_{n}=(\mathrm{ch}\beta_{n}x+\cos\beta_{n}x)-C_{n}(\mathrm{sh}\beta_{n}x+\sin\beta_{n}x),n\alpha 2, \end{array}\right.$$

where *C_n_* and *β_n_* are constants of the free beam function.

The differential equation of continuously supported concrete base is similar to Equation (21), and the second-order differential equation is directly given

(23) $${\ddot{Y}}_{n}(t)+\frac{C_{c}L_{c}}{M_{c}}{\dot{Y}}_{n}(t)+\frac{K_{c}+E_{c}I_{c}\beta_{n}^{4}L_{c}}{M_{c}}Y_{n}(t)=\sum_{g=1}^{N_{s}}\frac{F_{sg}(t)}{M_{c}}X_{n}(x_{g})$$

where *M*_c_, *E*_c_, *L*_c_, and *I*_c_ are the mass, modulus, length, and rotational inertia of the concrete base, respectively.

## 5. Comparative Analysis of the Vibration Reduction Effect and Evaluation Methods

For investigation into the influence of the different evaluation methods on the train-induced vibrations and the VRE of the FST, some calculation cases were designed, as shown in Table 6.

Case 1 in Table 6 is the basic case, in which the parameter adopts the 4 Hz secant stiffness within the load range required by the specification TJ/GW 121-2014. Case 2 further considers the frequency dependence of the secant stiffness. Because DIN 45673-7:2010 does not specify the frequency of the second preload and third preload tangent stiffness, the frequency-dependent tangent stiffness is used for comparative analysis in Cases 3 and 4. In Case 5, the modified FDPT model is adopted to reflect the preload change state of the STMs to accurately calculate insertion loss. Cases 1–5 shown in Table 6 were designed to explore the differences in existing evaluation methods so as to refine more accurate and suitable evaluation methods for different STMs. In this research, a six-car train with a speed of 160 km/h was used for simulation analysis to fully excite the vibrations of the DPFST. The track irregularity was modeled by the Association of American Railroads 5th class spectra, with wavelengths of 0.1−30 m.

The VRE is usually evaluated by the insertion loss Δ*L*_a_ between the DPFST and the monolithic track in the 1/3 frequency band from 1 to 200 Hz, defined as follows:

(24) $$\Delta L_{a}=10\mathrm{lg}\left(\sum_{i=1}^{n}10^{\frac{VL_{q}(i)}{10}}\right)-10\mathrm{lg}\left(\sum_{i=1}^{n}10^{\frac{VL_{h}(i)}{10}}\right)$$

*VL*_q_(*i*) and *VL*_h_(*i*) are the frequency division vibration level (dB) of the vertical vibration acceleration of the monolithic track and the DPFST at the *i*th center frequency in the 1/3 octave band. The frequency weightings in the 1/3 octaves refer to ISO 2361-1:1997 [38].

### 5.1. Vehicle Dynamic Model Evaluation of the Vibration Reduction Effect for STM-I

**Figure 10:**
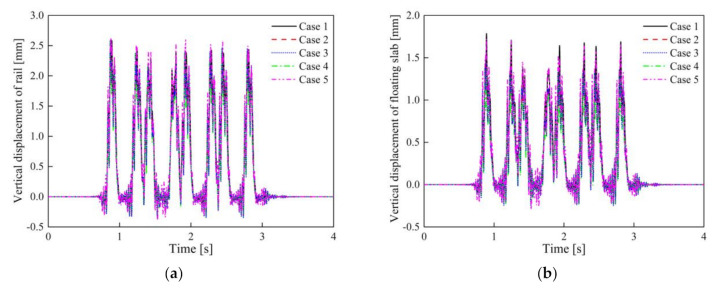
Vertical dynamic displacement: (**a**) rail; (**b**) slab in Cases 1–5.

To avoid repetition, the calculation results under the full load of the vehicle are illustrated. The vertical displacements of the rail and floating slab obtained by different evaluation methods in Cases 1–5 are shown in the Figure 10. It is clear that the dynamic displacement difference of the rail and floating slab in Cases 1–5 is very small. The calculation results (Cases 2–5) considering the frequency dependence of the STMs may be about 0.1 mm smaller in Case 1, but both meet the safety limit of 4 mm for rail and 3 mm for the floating slab [39].

**Figure 11:**
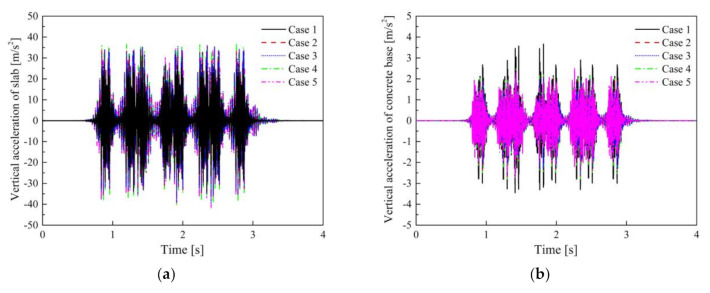
Vertical vibration acceleration: (**a**) slab; (**b**) concrete base in Cases 1–5.

**Figure 12:**
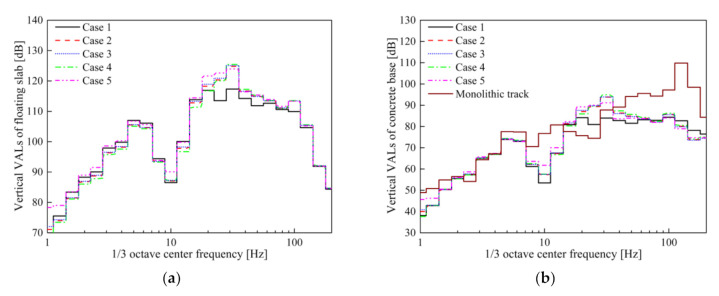
Vibration acceleration levels: (**a**) slab; (**b**) concrete base in Cases 1–5.

The time-domain vertical acceleration and vibration acceleration levels (VALs) of the slab with STM-I and concrete base in Cases 1–5 are shown in Figure 11 and Figure 12. The maximum vibration accelerations of the concrete base in Cases 1–5 are 3.52, 2.60, 2.52, 2.76, 2.71 m/s^2^, respectively. It can be found that the vibration acceleration of the slab without considering the frequency dependence of STM-I is the smallest among all the results, and the acceleration of the concrete base is the largest (i.e., Case 1). The time-domain acceleration results obtained by considering the frequency dependence of STM-I in Cases 2–5 show only minor differences.

It is evident from Figure 12 that the DPFST effectively suppresses vibration in almost the entire frequency band compared to the monolithic track, and yet the natural frequency vibration of the DPFST is amplified. In addition, the frequency-dependent characteristics of STM-I not only affect the vibration characteristics of the floating slab itself, but also amplify the vibration of the concrete base below 100 Hz, especially in the range of 20–31.5 Hz. The vibration acceleration results of the concrete base calculated by the KV model and the FDPT model are consistent with the conclusion of one study [16] in both the time and frequency domains.

In Case 1, due to the fact that the frequency- and preload-dependent characteristics of STM-I are not considered, the natural frequency of the floating slab is relatively low, which is mainly reflected in the value of 20 Hz. Although Cases 3 and 4 consider the influence of frequency characteristics, the stiffness under different preloads also has a slight difference in the vibration in the range of 20–31.5 Hz at the 1/3 octave frequency. The greater the stiffness of the STMs, the greater the vibration energy of the higher frequency within the resonant frequency range of the system. Since the stiffness of STM-I increases monotonically, compared with the second preload parameter (Case 3), the VALs of the slab obtained by the third preload parameter are slightly higher at the 1/3 octave frequency of 31.5 Hz and slightly lower at the 1/3 octave frequency of 20 Hz. When the preload dependence is further considered in the Case 5, the lower stiffness nonlinear segment between the first preload and second preload of STM-I is introduced into the calculation. Therefore, the VAL of the slab decreases at the 1/3 octave frequency of 31.5 Hz and increases at the 1/3 octave frequency of 20 Hz compared with Cases 3 and 4. The above influence is also reflected in the VALs of the concrete base, which is also the reason for the difference in the insertion loss calculated by different stiffness evaluation methods. The insertion losses obtained by different evaluation methods for STM-I under the conditions of empty load and full load are shown in Table 7.

Table 7 shows that the secant stiffness of STM-I does not consider the notion that the frequency dependence causes the insertion loss to be overestimated. Since the stiffness increases with the increasing frequency, the insertion loss calculated in the Case 2 using the frequency-dependent secant stiffness is smaller than in Case 1. The nonlinearity of STM-I is manifested as a monotonic increase in stiffness: the greater the preload selected by the evaluation method, the higher the frequency-dependent tangent stiffness and the smaller the insertion loss. Therefore, the evaluation effect of Case 3 is inferior to that of Case 4. Coincidentally, due to the weak nonlinearity of STM-I, the mechanical parameters used in the characterization of secant stiffness and tangent stiffness show minor differences, meaning the results of Cases 2 and 3 basically the same. Case 5 additionally considered the preload dependence of STM-I, the change of dynamic mechanical parameters with the axle load during operation reflects the process of stiffness from small to large, so the insertion loss is greater than Cases 3 and 4. Moreover, based on this feature of the FDPT model, Case 5 can accurately reflect the difference in the insertion loss caused by different axle loads. The insertion loss of Case 5 is reduced by 1.3 dB when the vehicle is fully loaded, while the attenuation of Cases 1–4 is the same—only 0.3 dB.

**Figure 13:**
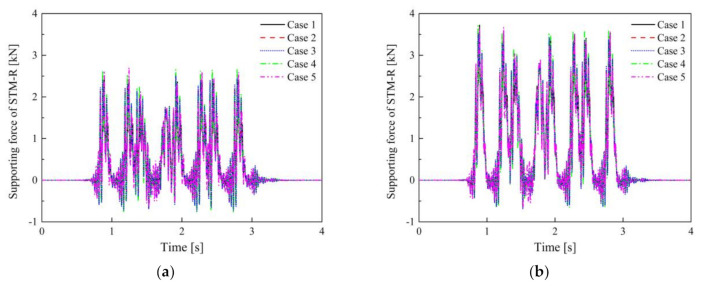
The supporting force of STM-I under (**a**) no load and (**b**) full load of the vehicle.

In contrast, the evaluation error in Case 4 is relatively large, regardless of whether the vehicle is empty or fully loaded, because the force that STM-I bears is less than, or has just reached, the third preload (see Figure 13). Therefore, using the third preload stiffness to evaluate the VRE is too conservative for STM-I, especially when the vehicle is under no load.

### 5.2. Vehicle Dynamic Model Evaluation of the Vibration Reduction Effect for STM-II

**Figure 14:**
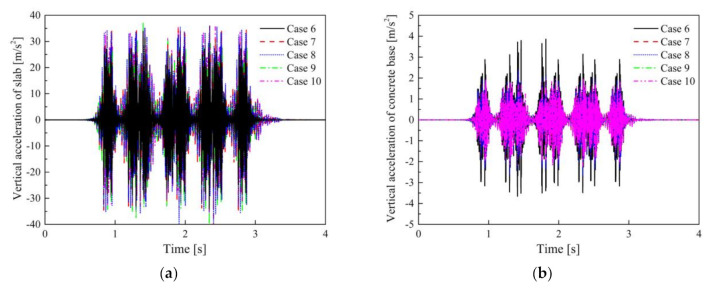
Vertical vibration acceleration: (**a**) slab; (**b**) concrete base in Cases 6–10.

**Figure 15:**
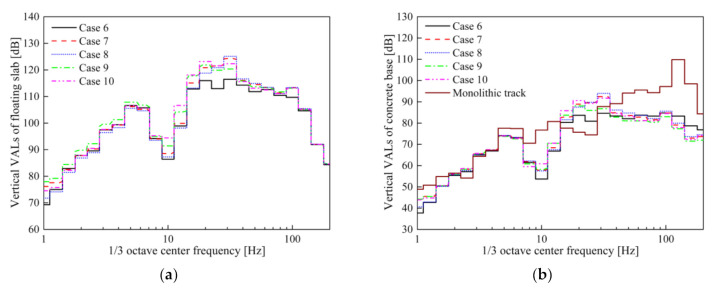
Vibration acceleration levels: (**a**) slab; (**b**) concrete base in Cases 6–10.

The subsequent sections focus on the differences in the VRE of the STMs obtained by different evaluation methods; thus, the figures of the dynamic displacement that meet the requirements of safe operation are omitted. The maximum dynamic displacement of the slab in Cases 6–10 under the full load of vehicle was 2.7 mm. The time-domain vertical acceleration and vibration acceleration levels (VALs) of the slab and concrete base in Cases 6–10 are shown in Figure 14 and Figure 15.

The maximum vibration accelerations of the concrete base in Cases 6–10 are 3.69, 2.27, 2.53, 1.95, and 2.18 m/s^2^, respectively. Similarly, when using STM-II, the impact of different evaluation methods on the DPFST is mainly reflected in the frequency band near the natural frequency, especially at the 1/3 octave frequency of 31.5 Hz. Since the stiffness of the third preload of STM-II is less than that of the second preload, the maximum frequency division vibration levels (*VL*_max_) of Cases 8 and 9 are mainly concentrated at 31.5 and 20 Hz, respectively. Compared with Case 9, the nonlinear segment with higher stiffness before the third preload is considered in Case 10; thus, the VAL at 31.5 Hz is higher than Case 9 but lower than that in Case 8 when only considering high stiffness. The insertion losses obtained by the different evaluation methods are shown in Table 8.

The effect of the frequency-dependent stiffness on the insertion loss is almost uniform for all of the STMs; thus, the insertion loss of Case 6 is the largest. The secant stiffness weakens the difference between different preloads of STM-II, meaning that the result of Case 7 is between that of Case 8 and Case 9. However, considering the nonlinear segment with the higher stiffness before the third preload, the actual VRE of Case 10 is less than that of Case 9. Moreover, unlike STM-I, the insertion loss of STM-II increases by 0.8 dB when the vehicle is fully loaded. This is because the increased load makes the lower stiffness segments of STM-II perform better. It can be concluded that the insertion loss error obtained by the third preload evaluation method is not negligible, but different from the evaluation for STM-I, and, thus, the VRE of STM-II will be overestimated.

### 5.3. Vehicle Dynamic Model Evaluation of the Vibration Reduction Effect for STM-III

**Figure 16:**
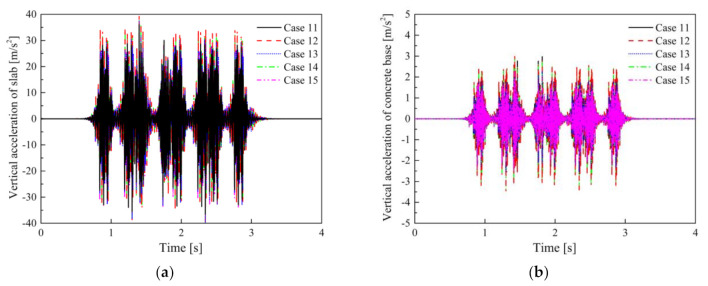
Vertical vibration acceleration: (**a**) slab; (**b**) concrete base in Cases 11–15.

The maximum dynamic displacements of the rail and slab in Cases 11–15 under the full load of vehicle are 2.6 and 1.8 mm respectively, which is basically the same as the results of STM-I and STM-II. Figure 16 shows the time-domain vertical acceleration of the slab and concrete base in Cases 11–15.

**Figure 17:**
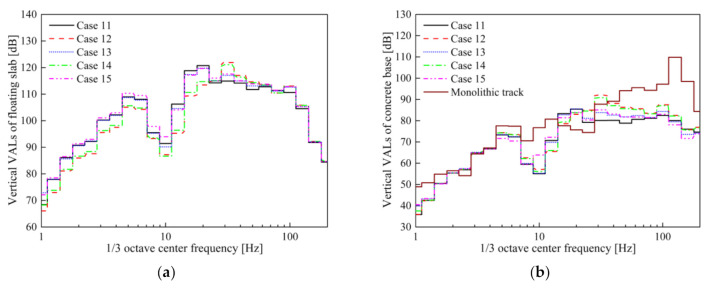
Vibration acceleration levels: (**a**) slab; (**b**) concrete base in Cases 11–15.

The maximum vibration accelerations of the concrete base in Cases 11–15 are 2.82, 3.52, 2.32, 3.12, and 2.45 m/s^2^, respectively. Despite the fact that the frequency dependence is considered in Cases 12–15, the true strong nonlinearity of STM-III results in a significant difference between the maximum vibration acceleration in Case 15 and the results of other evaluation methods (Case 12–14). The VALs of the slab and concrete base in Cases 11–15 are shown in Figure 17.

As described in Section 3.2, the frequency dependence effect of STM-III is more obvious, so the vibration in the wider frequency range of 20–100 Hz in the Cases 12–15 has a larger amplification compared with the Case 11. It is worth mentioning that the *VL*_max_ of Cases 12 and 14 are about 7 dB larger than Cases 13 and 15, which is very important for the insertion loss. Table 9 shows the insertion losses obtained by different evaluation methods for STM-III under the conditions of empty load and full load.

Compared with other STMs, the most significant difference between different evaluation methods for STM-III is that the frequency-dependent secant stiffness can no longer approximately reflect its true VRE. In addition, compared with STM-II, the third preloading did not overestimate its VRE of STM-III, but rather underestimated it. Another interesting finding is that the VRE of STM-III has little change in the no-load or full-load conditions, which is also what we hope to achieve under different axle loads. Compared with most STMs whose force–displacement curve is approximately linear (i.e., STM-I and STM-II), this type of STM with high-static–low-dynamic (HSLD) stiffness in the service load range is a good choice for improving the efficiency of vibration isolation.

### 5.4. Improvement of the Evaluation Methods

**Figure 18:**
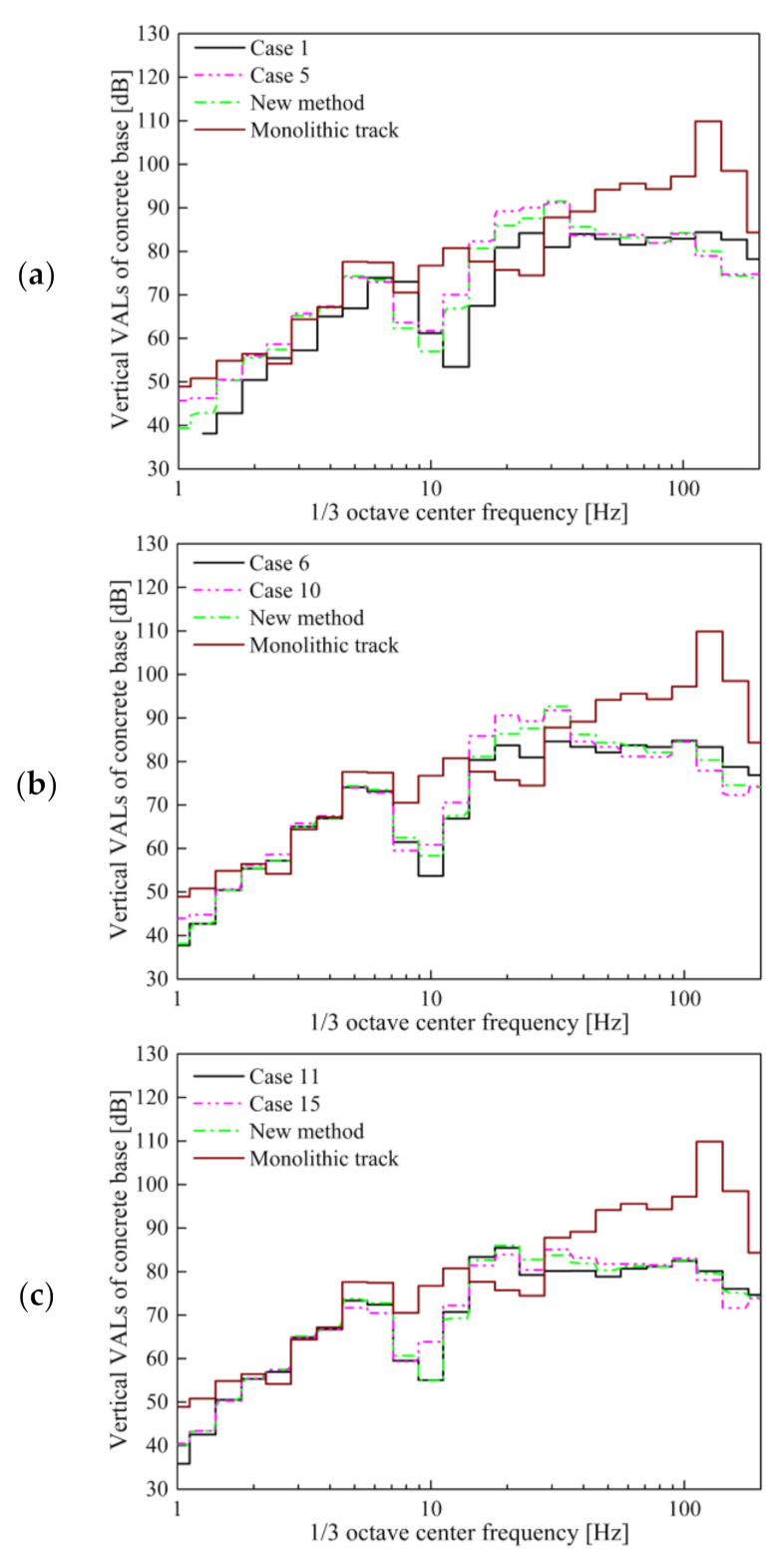
Vibration acceleration levels of the concrete base: (**a**) STM-I; (**b**) STM-II; (**c**) STM-III.

From the results of the above three STMs, it can be concluded that the insertion loss calculated by using the second preload frequency-dependent tangent stiffness is the closest to the real result. In order to facilitate its application, it is practical to adopt a single frequency parameter that can accurately reflect the vibration characteristics of the floating slab track and the VRE. Therefore, we proposed an improved calculation method for the second preload evaluation method, which uses the corresponding stiffness at the natural frequency to replace the frequency-dependent stiffness. It is worth mentioning that this natural frequency is not the tuning frequency under no load, but the natural frequency considering the P2 resonance during vehicle operation [40,41]. The stiffness used in the natural frequency calculation is the frequency-dependent stiffness of the second preloading, and the total mass is the sum of the mass of unsprung wheelsets and the mass of the FST system. Until the natural frequency is consistent with the frequency corresponding to the stiffness used in the calculation, the result is the true natural frequency of the DPFST under load [42]. The natural frequencies of the DPFST corresponding to the three types of STMs are 23.8, 24.2, and 20.0 Hz, and the stiffness corresponds to 0.0294, 0.0301, and 0.0211 N/mm^3^, respectively. As the vibration level of an empty vehicle is low and the vehicle is almost always in a loaded state, we evaluated the VRE of the vehicle under full load according to the method proposed in this article. The vertical VALs of the concrete base and insertion loss calculated by the new method for the three types of STMs are shown in Figure 18 and Table 10.

It is precisely because the parameters at the natural frequency can more accurately reflect the natural vibration characteristics of the DPFST that the VALs of the concrete base calculated by using the new method are relatively close to the actual results (especially the *VL*_max_ that has a greater impact on the insertion loss).

It is obvious that the maximum error between the result of the new method and the actual insertion loss calculated by considering the preload and frequency dependence is only 0.5 dB, which meets the error requirements of the engineering design. On the whole, this single parameter evaluation method at the natural frequency under the second preload is the most practical and accurate method at present.

## 6. Conclusions

A series of performance tests were conducted to study the preload dependence and frequency dependence of different STMs. The FDPT model was used instead of the KV model to accurately calculate the actual VRE obtained from the vehicle–DPFST coupled dynamic model. Next, the differences in the existing STM specifications in the evaluation of the VRE were analyzed. Finally, based on the above analysis, an accurate and practical single parameter evaluation method for guiding engineering design was proposed. The four main conclusions are as follows:

- In specification TJ/GW 121-2014 [30], the method of using the secant stiffness to evaluate the VRE must consider the influence of frequency-dependent characteristics of STMs; otherwise, the VRE will be significantly overestimated. The secant stiffness homogenizes the stiffness difference caused by different preloads in the service load range. For the STMs with monotonic stiffness, the evaluation error of this method decreases with the increased axle load. However, the homogenization effect of the secant stiffness has a relatively large evaluation error for the STMs with HSLD stiffness characteristics, because this evaluation method cannot reflect the nonlinear characteristics in the stiffness caused by load changes. The test method of the specification DIN 45673-7:2010 focuses on the difference under different preloads.
- The disadvantage of DIN 45673-7:2010 [31] is that in addition to not specifying which frequency stiffness is used to evaluate the VRE, the difference between the second preload and the third preload tangent stiffness in evaluating the VRE is not clarified. Moreover, with the tangent stiffness under a single preload, it is difficult to represent the actual mechanical state of the STMs changing with the axle load during the train operation. The insertion loss error obtained by the frequency-dependent tangent stiffness is reflected in the underestimation of the STMs with monotonically increased stiffness, the overestimation of the STMs with monotonically decreased stiffness, and the error increase with the increased preload used in the evaluation. The second preload frequency-dependent secant stiffness and the tangent stiffness have a small difference in the evaluation of the VRE of the STMs with monotonic stiffness, but only the frequency-dependent tangent stiffness is more accurate for the STMs with HSLD stiffness characteristics.
- Among the above different evaluation methods, the evaluation method using the second preload frequency-dependent stiffness of the DIN 45673-7:2010 [31] is the most accurate. However, for the convenience of engineering applications, it is most practical to use the second preload tangent stiffness at the natural frequency of the DPFST under the load to evaluate the VRE. The new method proposed can be used to guide the revision and improvement of the specification DIN 45673-7:2010 [31].
- In terms of the VRE of the different STMs, the insertion loss of STM-I with a monotonically increased stiffness decreases slightly with the increase in axel load, while the insertion loss of STM-II with a monotonically decreased stiffness improves with the increase in axel load. The VRE of STM-III with HSLD stiffness characteristics is both better and stable. This advantage of STM-III comes from the lower stiffness under the second preload. In addition, this also coincides with the evaluation and design idea of using the second preload tangent stiffness to evaluate the VRE.

## Figures and Tables

**Table 1:** Parameters of the finite element model.

Item	Parameter (Unit)	Magnitude	Element
Rail	Density (kg/m)	60.64	Beam4
	Young’s modulus (GPa)	206	
Fastener	Spacing (m)	0.60	Combin14
	Vertical stiffness (kN/mm)	35	
STM	Spacing (m)	0.05	Combin14
	Vertical stiffness (N/mm^3^)	0.01–0.05	
Floating slab	Length (m)	4.8	
	Width (m)	2.5	
	Thickness (m)	0.3	Solid45
	Modulus (GPa)	36	
	Density (kg/m^3^)	2500	
Applied load	Spacing (m)	2.5	
	Wheelset axle load (t)	17	-

**Table 2:** Test cases of the STMs.

Case	Preload	Excitation Method	Excitation Amplitude	Dynamic Frequency
1	From 0 to 5 kN at an interval of 0.5 kN	Force control	0 kN	0
2	From 0 to 5 kN at an interval of 1 kN	Velocity control	7 mm/s	1, 5, 10, 20, 40, 80, 100 Hz
3	2.5 kN	Force control	1.65 kN	1, 5, 10, 20, 40, 80, 100 Hz

**Table 3:** Four fitting parameters for static nonlinear characteristics.

Type	*a*_1_	*a*_2_	*a*_3_	*a*_4_
STM-I	0.094	−0.572	1.304	0.725
STM-II	0.256	−1.323	1.381	1.848
STM-III	−0.171	1.463	−3.398	2.936

**Table 4:** Fitting parameters in the FDPT model.

Type	*K*_2_′ (N/mm^3^)	*r*	*τ* (s)	*γ*
STM-I	0.0146	0.0853	3 × 10^−4^	0.26
STM-II	0.0175	0.0699	6 × 10^−5^	0.28
STM-III	0.0245	0.0610	7 × 10^−4^	0.45

**Table 5:** Parameters of the finite element model.

Item (Unit)	Value
Length between truck centers (m)	15.7
Vehicle body mass (t)	25.7/57.2
Bogie mass (t)	4.84
Wheelset mass (t)	1.89
Vehicle body moment (kg·m^2^)	1.4 × 10^6^
Bogie moment (kg·m^2^)	2.1 × 10^3^
Primary suspension stiffness (kN/m)	1.3 × 10^3^
Primary suspension damping (kN·s/m)	1.0 × 10^1^
Secondary suspension stiffness (kN/m)	2.2 × 10^2^
Secondary suspension damping (kN·s/m)	2.0 × 10^1^

**Table 6:** Calculation cases of the frequency and preload dependence.

Type	Case	Preload (kN)	KV Model (N/mm^3^)	FDPT Model				
				*K*_1_ (N/mm^3^)	*K*_2_′ (N/mm^3^)	*r*	*τ* (s)	*γ*
STM-I	1	0.8–4.1	0.0230	‒	‒	‒	‒	‒
	2	0.8–4.1	‒	0.0195	0.0345	‒	3 × 10^−4^	0.26
	3	2.5	‒	0.0182	0.0328	‒	3 × 10^−4^	0.26
	4	4.1	‒	0.0221	0.0319	‒	3 × 10^−4^	0.26
	5	0.8–4.1	‒	*K*_1_(*P*) in Table 4	0.0146	0.0853	3 × 10^−4^	0.26
STM-II	6	0.8–4.1	0.0243	‒	‒	‒	‒	‒
	7	0.8–4.1	‒	0.0198	0.0342	‒	6 × 10^−5^	0.28
	8	2.5	‒	0.0221	0.0369	‒	6 × 10^−5^	0.28
	9	4.1	‒	0.0142	0.0368	‒	6 × 10^−5^	0.28
	10	0.8–4.1	‒	*K*_2_(*P*) in Table 4	0.0175	0.0699	6 × 10^−5^	0.28
STM-III	11	0.8–4.1	0.0170	‒	‒	‒	‒	‒
	12	0.8–4.1	‒	0.0222	0.0480	‒	7 × 10^−4^	0.45
	13	2.5	‒	0.0088	0.0512	‒	7 × 10^−4^	0.45
	14	4.1	‒	0.0185	0.0520	‒	7 × 10^−4^	0.45
	15	0.8–4.1	‒	*K*_3_(*P*) in Table 4	0.0245	0.0610	7 × 10^−4^	0.45

**Table 7:** Insertion loss of the FST with STM-I under different load conditions (dB).

Axle Load	Case 1	Case 2	Case 3	Case 4	Case 5
Empty load	17.5	13.4	13.4	12.8	15.3
Full load	17.2	13.2	13.1	12.5	14.0

**Table 8:** Insertion loss of the FST with STM-I under different load conditions (dB).

Axle Load	Case 6	Case 7	Case 8	Case 9	Case 10
Empty load	17.8	14.1	13.4	16.9	12.8
Full load	17.5	13.8	13.1	16.6	13.7

**Table 9:** Insertion loss of the FST with STM-I under different load conditions (dB).

Axle Load	Case 11	Case 12	Case 13	Case 14	Case 15
Empty load	19.1	14.4	18.1	15.1	18.4
Full load	18.7	14.0	17.7	14.7	18.2

**Table 10:** Comparison of the insertion loss of the optimization method (dB).

Type	New Method	Modified FDPT Model
STM-I	14.5	14.0
STM-I	14.2	13.7
STM-III	17.8	18.2

## Data Availability

The data presented in this study are available on request from the corresponding author.
